# Childhood and recent maternal adverse experiences and mother-infant attachment influence early newborns’ neurobehavioural profiles

**DOI:** 10.1192/j.eurpsy.2022.389

**Published:** 2022-09-01

**Authors:** N. San Martín, Á. Castro Quintas, M. Daura-Corral, L. Marques Feixa, E. Eixarch, F. Crispi, L. De La Fuente Tomas, M.P. Garcia-Portilla, L. Fañanas

**Affiliations:** 1 University of Barcelona, Department Of Evolutive Biology, Ecology And Ambiental Sciences, Barcelona, Spain; 2 Centre for Biomedical Research Network on Mental Health (CIBERSAM), Instituto De Salut Carlos Iii, Madrid, Spain; 3 University of Barcelona, Evolutionary Biology, Ecology And Environmental Sciences, Barcelona, Spain; 4 University of Barcelona, Beeca, Barcelona, Spain; 5 Maternitat Hospital Clinic Barcelona, Bcnatal Fetal Medicine Research Center, Barcelona, Spain; 6 University of Oviedo, Department Of Psychiatry, Oviedo, Spain; 7 University of Oviedo, Department Of Psychiatry, oviedo, Spain

**Keywords:** Newborn, Neurodevelopment, Pregnancy, Maltreatment

## Abstract

**Introduction:**

Maternal stress during pregnancy influences fetal neurodevelopment, especially by the dysregulation of the HPA axis. However, less is known about whether maltreatment or stressful life experiences previous to pregnancy influence on developmental outcomes in the offspring.

**Objectives:**

To analyze newborns’ neurobehavioral profiles in a cohort of healthy pregnant women, according to 1) childhood and recent maternal adverse experiences and 2) mother-infant attachment.

**Methods:**

150 women were followed during the three trimesters of pregnancy. CTQ and AAT tests were employed to evaluate childhood and recent experiences of maltreatment, while infant and recent adverse experiences were evaluated using ETI-SR and SRSS, respectively. Newborns neurobehavioral profiles were defined at 8 weeks using the Neonatal Behavioral Assessment Scale (NBAS) and their temperament was assessed with IBQ. PBQ and PAI scales were employed to assess mother-infant attachment. A linear regression model was performed, adjusting for possible confounders.

**Results:**

Maternal childhood sexual abuse seems to be associated with greater difficulties in the newborns control of reactivity to external stimuli (β=0,517; *p-value=0.001*), while recent maternal stressful experiences are related to difficulties for states regulation (β=0,29; p-value=0,038). Regarding attachment, maltreated mothers tend to show ambivalent and avoidant styles. Interestingly, postnatal mother-infant attachment seems to modulate autonomous, motor and social-interactive abilities in the offspring (β=-0,227; p-value=0,033 // β=-0,329; p-value=0,006).

**Conclusions:**

Newborns from mothers exposed to maltreatment and negative life events previous to pregnancy show difficulties to organize and regulate the reactions to psychosocial stimuli. Future studies must disentangle whether maternal attachment style is a modulator of this association.

**Disclosure:**

No significant relationships.

